# Protocol for performing 3D-STORM-based nanoscale organization of NMDA receptors in hippocampal brain tissue

**DOI:** 10.1016/j.xpro.2025.103639

**Published:** 2025-02-24

**Authors:** Joana S. Ferreira, Jeanne Linarès-Loyez, Pierre Bon, Laurent Cognet, Laurent Groc

**Affiliations:** 1 Interdisciplinary Institute for Neurosciences - IINS, UMR 5297, Université de Bordeaux, 33076 Bordeaux Cedex, France; 2 Laboratoire Photonique Numérique et Nanosciences, UMR 5298, Université de Bordeaux, 33400 Talence, France; 3 Institut d’Optique & CNRS, LP2N UMR 5298, Talence, France; 4 Multidisciplinary Institute of Ageing – MIA-PORTUGAL, Center for Neuroscience and Cell Biology – CNC-UC, Centre for Innovative Biomedicine and Biotechnology – CIBB, Institute of Interdisciplinary Research – iiiUC, University of Coimbra, 3004-504 Coimbra, Portugal; 5 XLIM, UMR 7252, CNRS, Université de Limoges, 87000 Limoges, France

**Keywords:** Microscopy, Neuroscience

## Abstract

Direct stochastic optical reconstruction microscopy (dSTORM) unveils ionotropic N-methyl-D-aspartate receptor (NMDAR) organization into discrete nanometer-size domains (nanoclusters) within the postsynaptic density (PSD) of glutamatergic synapses, tuning receptor signaling. Here, we present a protocol to perform 3D-dSTORM imaging of the NMDAR in organotypic and acute rat hippocampal brain slices by combining conventional dSTORM with fluorescent self-interference. We describe steps for sample preparation, immunohistochemistry, 3D-dSTORM acquisition, and image analysis to successfully super-resolve NMDAR nanodomains in three dimensions.

For complete details on the use and execution of this protocol, please refer to Kellermayer et al.,^1^ Bon et al.,^2^ and Ferreira et al.^3^

## Highlights

• Steps to prepare hippocampal brain slices for single-molecule imaging
• Instructions for acute brain slice preparation from the rat brain after perfusion with PFA
• Guidance on imaging settings to super-resolve NMDA receptor nanodomains in 2D and 3D
• Analyze and compare the nanoscale organization of NMDA receptor subtypes in brain slices

## Abstract (editor highlights)

Publisher’s note: Undertaking any experimental protocol requires adherence to local institutional guidelines for laboratory safety and ethics.

## Graphical abstract

**Figure:**
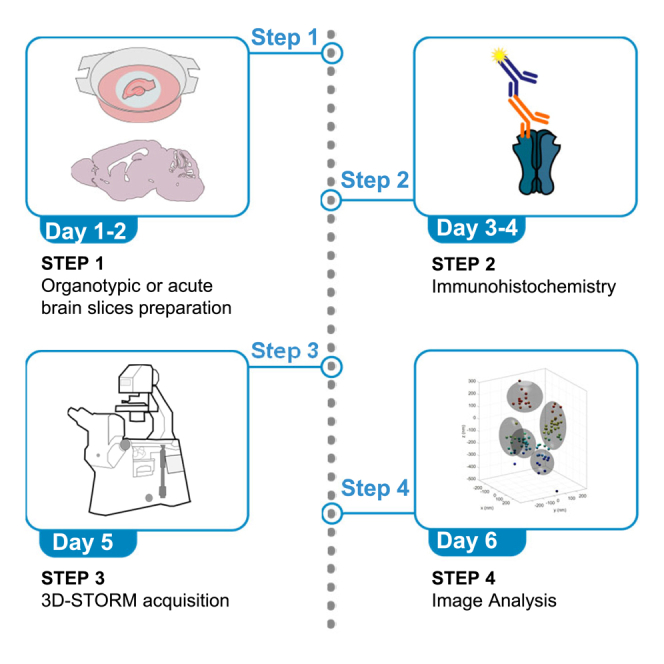

## Before you begin

The protocol describes the sample preparation and analysis for imaging N-methyl-D-aspartate receptor (NMDAR) nanodomains with 3D-dSTORM in brain tissue: organotypic cultured slices and acute brain slices. Nanodomains are defined as nanometer-size dense objects within clusters, which correspond to puncta of identical size to diffraction-limited microscopy puncta. We predict the presented method can be used for the 3D-dSTORM imaging of other surface proteins, namely other neurotransmitter receptors, given that specific antibodies are available and that they are highly abundant in the tissue.

This protocol builds upon our previous work using a combination of dSTORM microscopy with fluorescent self-interference (SELFI),^2^ to achieve the 3D position of the emitter and show that the nanoscale organization of NMDAR is dependent on the receptor’s subunit composition^1^ and distance-to-soma,^3^ using cultured primary neurons. In this work,^1^ we compared the 3D clustering with the corresponding 2D xy (in plane) projection and demonstrated that 3D-dSTORM captures on average only one nanodomain per cluster more than the respective 2D projection.^1^ The number of nanodomains per cluster identified with 3D-dSTORM was representative of the diversity of nanoscale organizations and the differences found between the number of nanodomains within GluN2A- or GluN2-NMDAR clusters was comparable to the number of nanodomains captured by conventional 2D-dSTORM.^1^^,^^3^^,^^4^^,^^5^

### Institutional permissions

Animal procedures were conducted following the European Community guidelines (Directive 2010/63/EU) regulating animal research, and were approved by the local Bordeaux Ethics Committee (APAFIS#3420-2015112610591204). Investigators utilizing this protocol must acquire the relevant permissions for working with animals from their institution and their relevant governing body.

### 4% PFA–4% sucrose preparation

**Timing: 1–2 h**

1. For the slice fixation:
  a. Use one aliquot of 3 mL 16% PFA – 16% sucrose stock solution.
  b. Add 12 mL of PBS 1X.
  c. Bring to RT before use.

### Neurobasal medium–1% BSA preparation

**Timing: 5 min**

2. For primary antibody incubation of organotypic brain slices prepare.
  a. 100 μL per slice of neurobasal medium (NB).
  b. Add 1% (w/v) of BSA.

**CRITICAL:** Always prepare fresh.

### Immunostaining solution preparation

**Timing: 30 min**

3. Prepare 150 μL of PBS – NH_4_Cl for each organotypic slice incubation.
  a. PBS 1x.
  b. 50 mM NH_4_Cl.

**CRITICAL:** NH_4_Cl is harmful if swallowed. Causes serious eye irritation.

***Optional:*** Prepare a bigger volume and store it at room temperature (RT, 20°C–25°C) for ≤ 6 months.

4. Prepare 500 μL per slice of PBS – Triton for permeabilization and washes of acute slices.
  a. PBS 1x.
  b. 0.3% of Triton (volume/volume).

**CRITICAL:** Triton is harmful if swallowed, causes skin irritation and serious eye damage. Very toxic to aquatic life with long-lasting effects.

***Optional:*** Prepare a bigger volume and store it at room temperature (RT, 20°C–25°C) for ≤ 6 months.

5. Prepare 500 μL of PBS – Triton – 5% BSA per slice for blocking of acute slices.
  a. PBS 1x.
  b. 0.3% of Triton (v/v).
  c. 5% of BSA (w/v).

**CRITICAL:** Always prepare fresh.

6. Prepare 200–250 μL of PBS – Triton – 1% BSA per slice for secondary antibodies incubation of acute slices.
  a. PBS 1x.
  b. 0.3% of Triton (v/v).
  c. 1% of BSA (w/v).

**CRITICAL:** Always prepare fresh.

7. Prepare 300–500 μL per slice of PBS – Triton – 3% BSA – Gelatin for blocking, permeabilization, and incubation with secondary antibodies of organotypic slices.
  a. PBS 1x.
  b. 3% of BSA (w/v).
  c. 0.1% of gelatin (v/v).
    **Caution:** Bring Gelatin to RT before solution preparation.
  d. 0.3% Triton (v/v).
    **CRITICAL:** Always prepare fresh.

### Switching buffer preparation

8. Mix.
  a. 1 mL glucose stock solution.
  b. 120 μL enzyme stock solution.
  c. 120 μL MEA stock solution.
  d. Adjust pH to 7.8–8.0 if necessary.

**CRITICAL:** Always use fresh aliquots of each component before each imaging session and avoid bubbles when mixing.

## Key resources table

**Table:** 

REAGENT or RESOURCE	SOURCE	IDENTIFIER
**Antibodies**		

Anti-GluN1, dilution 1:500	Lü et al.^6^; Kellermayer et al.^1^	N/A
Anti-GluN2A, dilution 1:200	Kellermayer et al.^1^	N/A
Anti-GluN2B, dilution 1:200	Kellermayer et al.^1^	N/A
Anti-rabbit Alexa Fluor 647, dilution 1:500	Thermo Fisher Scientific	Cat# A-21244; RRID: AB_2535812
Anti-mouse Alexa Fluor 647, dilution 1:500	Thermo Fisher Scientific	Cat# A-21235; RRID: AB_2535804

**Biological samples**		

Organotypic hippocampal brain slices	In this paper	N/A
Acute brain slices	In this paper	N/A

**Chemicals, peptides, and recombinant proteins**		

Ammonium chloride (NH4Cl, ReagentPlus, ≥99.5%)	Sigma-Aldrich	A4514; CAS: 12125-02-9
Bovine serum albumin (BSA, heat shock fraction, protease-free, essentially globulin free, pH 7, ≥98%)	Sigma-Aldrich	A3059; CAS: 9048-46-8
Calcium chloride solution (CaCl_2_, 1 M)	Sigma-Aldrich	21115; CAS: 10043-52-4
Catalase from bovine liver (aqueous suspension, 40,000–60,000 units/mg protein)	Sigma-Aldrich	C100; CAS: 9001-05-2
Cysteamine hydrochloride (MEA-HCL, HSCH_2_CH_2_NH_2_.HCl)	Sigma-Aldrich	M6500; CAS: 156-57-0
D-(+)-glucose (C_6_H_12_O_6_)	Sigma-Aldrich	G6152; CAS: 50-99-7
D-(+)-glucose solution (45% in H_2_O)	Sigma-Aldrich	G8769; CAS: 50-99-7
EXAGON solution injectable	Axience SAS	
Gibco Basal Medium Eagle (BME)	Thermo Fisher Scientific	41010026
Gibco Hank’s balanced salt solution (HBSS), calcium, magnesium, no phenol red	Thermo Fisher Scientific	14025050
Gibco Neurobasal medium, minus phenol red	Thermo Fisher Scientific	12348017
Gibco HEPES (N-2-hydroxyethylpiperazine-N-2-ethane sulfonic acid) (1 M)	Thermo Fisher Scientific	15630056
Gibco Horse Serum, heat-inactivated, New Zealand origin	Thermo Fisher Scientific	26050088
Gibco B-27 Plus Supplement (50X)	Thermo Fisher Scientific	A3582801
Glycerol	Sigma-Aldrich	G5516; CAS: 56-81-5
Pyranose oxidase from Coriolus sp.	Sigma-Aldrich	P4234; CAS: 37250-80-9
Gelatin from cold water fish skin (40%–50% in H_2_O)	Sigma-Aldrich	G7765; CAS: 9000-70-8
LUROCAÏNE	Vetoquinol	
Magnesium chloride solution (MgCl_2_, 1 M)	Sigma-Aldrich	63069; CAS: 7786-30-3
Magnesium sulfate heptahydrate (MgSO_4_.7H_2_O)	Sigma-Aldrich	M9397; CAS: 10034-99-8
Sodium hydroxide (NaOH, reagent grade, ≥98%, pellets, anhydrous)	Sigma-Aldrich	S5881; CAS: 1310-73-2
Hydrochloric acid (HCl, ACS reagent, 37%)	Sigma-Aldrich	320331; CAS: 7647-01-0
Paraformaldehyde (PFA, powder)	VWR Chemicals	28794.295; CAS: 30525-89-4
Phenol red solution (liquid, 0.5%, sterile-filtered, BioReagent, suitable for cell culture)	Thermo Fisher Scientific	P0290; CAS: 143-74-8
Phosphate buffer saline solution (PBS 10X, pH∼7.0)	Euromedex	ET330
Potassium chloride (KCl, ReagentPlus, ≥99.0%)	Sigma-Aldrich	P4504 ; CAS: 7447-40-7
Potassium phosphate monobasic (KH2PO4, ReagentPlus, ≥99.0%)	Sigma-Aldrich	P5379; CAS: 7778-77-0
Sodium bicarbonate (NaHCO_3_, ACS reagent, ≥99.7%)	Sigma-Aldrich	S6014; CAS: 144-55-8
Sodium chloride (NaCl, BioXtra, ≥99.5%)	Sigma-Aldrich	S7653; CAS: 7647-14-5
Sodium phosphate dibasic dodecahydrate (Na_2_HPO_4_.12H_2_O, BioXtra, ≥99.0%)	Sigma-Aldrich	71649; CAS: 10039-32-4
Sucrose (C_12_H_22_O_11_, BioUltra, for molecular biology, ≥99.5%)	Sigma-Aldrich	84097; CAS: 57-50-1
Thermo ScientificGlutaMAX supplement	Thermo Fisher Scientific	35050038
Tris(2-carboxyethyl)phosphine hydrochloride (TCEP, C_9_H_15_O_6_P.HCl)	Sigma-Aldrich	C4706; CAS: 51805-45-9
Triton X-100	Sigma-Aldrich	T9284; CAS: 9036-19-5
Trizma hydrochloride (Tris-HCl, BioPerformance certified, suitable for cell culture, ≥99.0%)	Sigma-Aldrich	T5941; CAS: 1185-53-1

**Experimental models: Organisms/strains**		

*Rattus norvegicus*, wild type, post-natal day (P) 5 to P7, males and females	Janvier Labs	Sprague-Dawley RjHan:SD
*Rattus norvegicus*, wild type, P15–P37, male	Janvier Labs	Sprague-Dawley RjHan:SD

**Software and algorithms**		

SR-Tesseler	Levet et al.^7^	https://github.com/flevet/SR-Tesseler
labVIEW	National Instruments	

**Other**		

BioLite cell culture-treated dishes	Sigma-Aldrich	130181
BOCHEM Double spatula flexible total length 180 mm	Dutscher	076209
Cell Culture Multiwell Plate, 12 Well, Ps, Clear, Cellstar®, Tc, Lid with Condensation Rings, Sterile, Single Packed	Greiner Bio-One	665180
CO_2_ Incubator Thermo Scientific HERACELL VIOS 160	Sigma-Aldrich	51030434
CytoOne Dish 35 × 10 mm, TC-treated	CytoOne dishes	CC7682-3340
CytoOne Dish, TC-treated (diameter 35 mm)	CytoOne dishes	CC7682-3340
Dumont #4 forceps, straight	Fine Science Tools – FST	11241-30
Dumont #7 - fine forceps, curved	Fine Science Tools – FST	11274-20
Falcon 6-well clear flat bottom TC-treated multiwell cell culture plate, with lid, individually wrapped, sterile	Corning	353046
Leica CM3050 S Cryostat	Leica Biosystems	N/A
Leica VT1000 S Vibrating blade microtome	Leica Biosystems	N/A
McIlwain tissue chopper MTC/2E	N/A	N/A
Millicell cell culture insert (diameter 30 mm, hydrophilic PTFE, 0.4 μm pore size)	Millipore	PICM03050
Omnipore membrane filter (0.45 μm pore size, hydrophilic PTFE membrane, 25 mm diameter)	Millipore	JHWP02500
Stereo Microscope Lighting SCHOTT KL 1500 LCD		
Stereo Microscope Nikon SMZ445	Nikon	
Stericup-GP sterile vacuum filtration system	Sigma-Aldrich	S2GPU05RE
Surgical scissors – sharp	Fine Science Tools – FST	14002–12
Thermo Scientific BioLite cell culture-treated dishes (diameter 60 mm)	Thermo Fisher Scientific	130181
VAPRO Vapor Pressure Osmometer Model 5600		
Peristaltic perfusion pump		
Cannula (1.4 mm diameter) for intra-ventricular perfusion		
Imaging spacer, double adhesive sides, Grace Bio-Labs SecureSeal imaging spacer, one well, 20 mm diameter, 0.12 mm deep	Merck	GBL654006-100EA
Ludin Chamber type 1		
Plain microscope slides (1 mm thick)	VWR	HECH4240101

## Materials and equipment

### 4% PFA

• Dissolve 80 g of PFA in 1 L of filtered H2O, add 20 mL of 1 M NaOH, and stir gently at approximately 60°C until PFA is dissolved.

**CRITICAL:** Temperature should not exceed 70°C.

**Caution:** PFA is extremely toxic, wear an FFP3 mask when working with the powder.

• Let the PFA solution cool down.
• Prepare the PBS buffer, by adding 11.2 g of KH_2_PO_4_, and 71.6 g of Na_2_HPO_4_.12H_2_O into 950 mL of filtered H2O.
• Mix the PFA solution with the PBS.
• Adjust the pH to 7.4 and the volume to 2 L.
• Store at 4°C for ≤1 month.

### 16% PFA–16% sucrose

• Dissolve 64 g of PFA in 100 mL of filtered H_2_O, add 2 mL of 1 M NaOH, and stir gently at approximately 60°C until PFA is dissolved.

**CRITICAL:** Temperature should not exceed 70°C.

**Caution:** PFA is extremely toxic, wear an FFP3 mask when working with the powder.

• Once the PFA is dissolved, let the solution cool down and add 20 mL of PBS 10X.
• Adjust the pH to 7.4 and the volume to 200 mL.
• Prepare 3 mL aliquots in 15 mL tubes and store them at – 20°C.

**Table:** Culture medium

Reagent	Final concentration	Amount
BME	N/A	200 mL
HBSS	N/A	100 mL
Glucose Solution 45%	N/A	4 mL
GlutaMAX	N/A	2 mL
Horse Serum	N/A	100 mL
**Total**	**N/A**	**400 mL**

Store at 4°C for ≤ 3 weeks.

**CRITICAL:** All mediums should be prepared under sterile conditions.

**Table:** Dissection medium 10X

Reagent	Final concentration	Amount
CaCl_2_ (1 M)	5 mM	2.5 mL
KCl	25 mM	932.5 mg
KH_2_PO_4_	6.6 mM	450 mg
MgCl_2_ (1 M)	20 mM	10 mL
MgSO_4_.7H_2_O	2.8 mM	350 mg
NaCl	0.5 M	16.61 g
Na_2_HPO_4_.12H_2_O	8.5 mM	1.525 g
Glucose	250 mM	22.52 g
**Total**	**N/A**	**500 mL**

• Adjust the final volume with filtered H_2_O and filter the solution.
• Prepare 50 mL aliquots at store at −20°C, for ≤ 3 months.

**CRITICAL:** All mediums should be prepared under sterile conditions.

**Table:** Dissection medium 1X

Reagent	Final concentration	Amount
Dissection medium 10X	5 mM	50 mL
Sucrose	175 mM	30 g
NaHCO_3_	2.7 mM	113 mg
Phenol red solution	N/A	1 mL
HEPES (1 M, pH 7.3)	2 mM	1 mL
**Total**	**N/A**	**500 mL**

• Expected osmolarity 330 mOsm.
• Adjust the volume with filtered H2O and filter the solution.
• Store at 4°C for ≤ 3 weeks.

**CRITICAL:** All mediums should be prepared under sterile conditions.

### Enzyme stock solution

• Prepare the enzyme base solution.

**Table:** 

Reagent	Final concentration	Amount
Glycerol	50%	25 mL
KCl (1 M)	25 mM	1.25 mL
Tris-HCl (1 M), pH 7.5	20 mM	1 mL
ddH2O	N/A	22.5 mL
**Total**	**N/A**	**50 mL**

• Prepare 10 mL aliquots and store at −20°C, for ≤ 3 months.
• Prepare the enzyme solution.

**Table:** 

Reagent	Final concentration	Amount
Enzyme base solution	N/A	10 mL
Catalase	N/A	20 μL
TCEP (1 M)	4 mM	40 μL
Pyranose oxidase	N/A	10 mg
**Total**	**N/A**	**10 mL**

• Prepare 250 μL aliquots and store at −20°C, for ≤ 3 months.

**CRITICAL:** Pyranose oxidase may cause allergy or asthma symptoms or breathing difficulties if inhaled. TCEP causes severe skin burns and eye damage.

**Table:** Glucose stock solution

Reagent	Final concentration	Amount
Glucose	0.5 M	5 g
Glycerol	10%	5 mL
ddH2O	N/A	45 mL
**Total**	**N/A**	**50 mL**

• Prepare 1000 μL aliquots and store at −20°C, for ≤ 3 months.

### MEA (2 M)

• Dissolve 1.136 g of MEA-HCl in 10 mL distilled H_2_O.
• Prepare 250 μL aliquots and store at −20°C.

**CRITICAL:** Harmful if swallowed, may cause an allergic skin reaction and/or respiratory irritation, causing serious eye irritation.

**Table:** Neurobasal medium (NB)

Reagent	Final concentration	Amount
Neurobasal Medium	N/A	100 mL
B-27 Plus Supplement	N/A	2 mL
GlutaMAX 100x Supplement	0.5 mM	250 μL

• Prepare 10 mL aliquots and store at 4°C for ≤ 3 weeks.

**CRITICAL:** All mediums should be prepared under sterile conditions.

### Microscope setup

The imaging system was constructed around an inverted z-motorized NikonTiE (Nikon) microscope equipped with a silicone immersion objective 60×NA = 1.3 (Nikon). Fluorophores were excited by a 638 nm laser diode (HL63193, Oclaro) in wide-field epi-configuration (from 100 mW to 500 mW over 30 × 30 μm^2^) while the transmission white light was emitted by a halogen lamp (Nikon) filtered at 561 ± 10 nm (Semrock band-pass filter), distinct from the excitation and the emission fluorescence bands. Transmission light was acquired in real time with a quantitative phase imaging module (QPI, SID4-element, Phasics) to perform 3D microscope drift measurements. Blinking of the Alexa Fluor 647 dyes was reactivated by 405 nm illumination (Intensilight, Nikon, Japan, using a 395/20 nm Semrock band-pass filter).

The 3D localization method is the SELFI method based on the self-interferences of the light collected and detected from each fluorescent molecule, creating pattern within each point spread function that can be used (thanks to signal processing in the numerical Fourier space) to determine the lateral and axial super-localization. The signal for the axial localization (i.e., interference pattern period modulation) arises from the wavefront curvature directly linked to the axial position of the fluorescence molecule image. The lateral localization is obtained with conventional 2D Gaussian fitting of the point spread function. The SELFI fluorescence detection path was composed of a phase-only diffraction grating (custom-made but available as Phimask, from Idylle-Labs) inserted close to the imaging plane of the microscope output port and optically transported by an imaging relay (2x, MML2, MORITEX) on an sCMOS sensor (Orca Flash 4, Hamamatsu). The SELFI module was used to generate and record single fluorescent emitter interference images.

The lateral and axial localization precision are both primarily linked to the number of detected photons with respect to the photon background (called signal to noise ratio) and the point-spread function size. The localization precision is then affected by the defocus with respect to the objective focal plane. The shape and the ratio between axial and lateral resolution remain identical when the signal to noise ratio is changing. The local aberrations are also affecting the resolution but the effect is more robust than classical 3D localization methods based on point spread function shaping.

The resolution is affected by multiple parameters (in order of importance): the localization precision, the encumbrance and density of the labeling, and the sample drifts. We can neglect the last one since an active 3D stabilization is used with precision much higher than the other effects affecting the resolution. The resolution is measured using 3D Fourier ring correlation for each observed region and the values are of about 20–30 nm in xy and 40–60 nm in z. Localization precisions.

## Step-by-step method details

### Organotypic slice preparation

**Timing: 1 h 30 min to 2 h**

**Figure 1:**
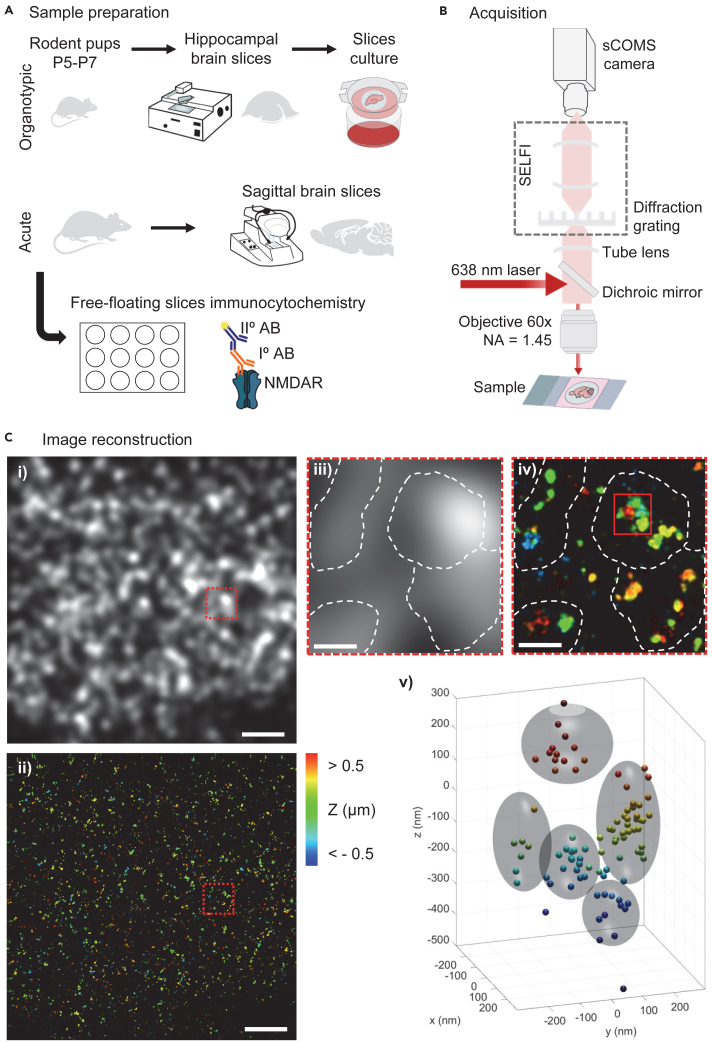
Experimental pipeline (A) Schematic of sample preparation main steps to obtain i) organotypic brain slices or ii) acute brain slices, which are then immunostained with specific primary and secondary antibodies, and mounted in an imaging chamber for 3D-dSTORM. (B) Microscopy setup for 3D-dSTORM imaging. (C) Image reconstruction of GluN2A-NMDAR organotypic slice acquisition. Z-depth 20 μm i) epifluorescence image of GluN2A-NMDAR. Scale 5 μm. ii) corresponding super-resolved image of GluN2A-NMDAR clusters. Scale 5 μm. iii) magnification of GluN2A-NMDAR epifluorescence clusters. Dash line defines four distinct clusters. Scale 1 μm. iv) magnification of the corresponding GluN2A-NMDAR super-resolved clusters. Scale 1 μm. v) 3D-super-resolved reconstruction of one GluN2A-NMDAR. The gray sphere defines five distinct nanodomains.

This step describes the organotypic slice preparation from the hippocampus of rat postnatal day (P) 5 to P7 rat pups and how to maintain them in culture for 10–12 days *in vitro* (div)^8^^,^^9^ (Figure 1A).

1. Prepare the culture inserts.
  a. Cut the FHLC membrane into approximately 5 mm pieces in a triangular or rectangular shape (enough to fit one slice).
  b. Place 4 to 6 inserts per cell culture insert, previously placed into a well from a 6 multi-well plate containing 1 mL of culture medium.

**CRITICAL:** This step should be done a minimum of two hours before dissection.

2. Hippocampal dissection.
  a. Remove the brain from P5-P7 rat pups, by dissecting the head and carefully opening their skull.
  b. Place the brain in a 60 mm Petri dish, containing 1–2 mL of dissection medium, and remove the brainstem, under the binocular magnifier.
  c. Separate the two hemispheres, remove the meninges, and dissect the hippocampus.
  d. Place the two hippocampi side by side, perpendicular to the blade, on the tissue chopper stage.
  e. Prepare 350 μm thick slices.
3. Slices platting.
  a. Use a 1000 mL micropipette tip to aspirate the slices and let them rest in a 35 mm Petri dish containing 2 mL of dissection medium for 25 min.
  b. Under the binocular magnifier separate the slices using a spatula, aspirate each slice individually, and place each slice in an individual insert in the multi-well plate containing the culture medium.

**CRITICAL:** Once you aspirate the slice let it deposit at the bottom of the micropipette tip before platting the slice into the insert. Avoid expelling a big volume of dissection medium, this will cause your slice to slide outside of the insert.

4. Place the organotypic slices in a 35°C / 5% CO2 incubator.
5. Change the culture medium every two to three days with fresh pre-warmed medium.

### Immunostaining for organotypic slices

**Timing: 2 days (steps 6–8: 3 h 30 min; steps 9–11: 6 h 30 min; steps 8 and 12: overnight [6–8 h])**.

This step describes the optimized protocol for immunostaining of NMDARs using primary antibodies against the extracellular N-terminal of the receptors (Table 1) and imaging by 3D-dSTORM (Figure 1A).

***Note:*** This protocol may be applied to the staining of other surface membranes.

**CRITICAL:** The specificity of primary antibodies should be validated using knockout samples (e.g. brain tissue from knock-out animals, knockout using shRNA, siRNA, or CRISPR-Cas9 approaches) or heterologous systems using exogenous expression of the specific subunits.^1^^,^^10^ Equally, the specificity of secondary antibodies should be evaluated by performing the protocol in the absence of the primary antibody.

6. Primary antibody incubation.
  a. After 10–12 div, remove individual slice inserts.
  b. Dip each slice in 300 μL per slice of NB – 1% of BSA, containing primary antibodies against the extracellular portion of the glutamate receptor subunit at the appropriate dilution (Table 1). Troubleshooting 1.
    ***Note:*** The antibodies against GluN subunits are either commercially available^4^^,^^6^^,^^11^ or upon request to the authors.
  c. Incubate for 2 h at 34°C.
7. Wash unbound antibodies.
  a. Remove the medium containing primary antibodies.
  b. Incubate 1 h with NB – 1% BSA (500 μL per slice) at 34°C. Troubleshooting 2.
8. Slices fixation.
  a. Transfer the slice to 300–500 μL of 4% PFA – 4% sucrose.
  b. Incubate overnight (ON – 6 to 8 h) at 4°C.
9. Wash unbound antibodies.
  a. Wash the slice two times with PBS (500 μL per slice), for 45 min to 1 h at room temperature (RT, 20°C–25°C). Troubleshooting 2.
10. Block non-specific binding.
  a. Incubate for 2 h the slice with PBS – Triton – 3% BSA – Gelatin (500 μL per slice) at RT. Troubleshooting 2.
11. Secondary antibody incubation.
  a. Incubate the slice with 300 μL of NB – 1% of BSA, containing secondary antibodies, with the appropriate dilutions (Table 1), for 3 h at RT. Troubleshooting 1.
12. Wash unbound antibodies.
  a. Wash in PBS ON at 4°C. Troubleshooting 2.

**CRITICAL:** Image in the following 2–3 days.

**CRITICAL:** Contrary to protocols for immunostaining for dSTORM of primary neuronal cultures do not perform a second fixation step, it may cause imaging artifacts.

**Table 1:** Primary and secondary antibodies

Antibody	Sequence target	Dilution
Anti-GluN1	Unavailable (extracellular, clone 10B11)	1:500
Anti-GluN2A	GHSHDVTERELRN (extracellular)	1:200
Anti-GluN2B	NTHEKRIYQSNMLNR (extracellular)	1:200
Anti-rabbit Alexa Fluor 647	N/A	1:500
Anti-mouse Alexa Fluor 647	N/A	1:500

### Acute slice preparation

**Timing: 1 day and a half (steps 13–15: 4 h; step 16: overnight [6–8 h]; step 17: 30 min to 2 h)**

This step describes the acute brain slice preparation from the rat brain P15–P37 rat brains after perfusion with PFA^12^^,^^13^ (Figure 1A).

13. Prepare a pentobarbital/lidocaine mixture.
  a. Dilute 0.25 mL of EXAGON and 0.5 mL of LUROCAINE in 2.6 mL of water for injection.

**CRITICAL:** Do not store.

14. Anesthetize the animals P15-P35.
  a. Inject the solution intraperitoneally (2 mL for 300 g of body weight).

**CRITICAL:** Check the toe-pinch reflex of the animal before initiating any procedure.

15. Perfusion.
  a. Perfuse transcardially with a 0.9% NaCl solution followed by 4% PFA, with a flow rate of about 20 mL/min for 300 g of body weight.
16. Decapitate the head and remove the brain, and store for 24 h in 4% PFA.

***Optional:*** The brain can be subsequently stored in PBS at 4°C, until the following step.

17. Prepare 120 μm sagittal slices in the vibratome.
18. Store in PBS at 4°C.

### Immunostaining for acute slices

**Timing: 1 day and a half (steps 19–21: 2 h 30 min; steps 22–25: 4 h; step 21: overnight [6–8 h]).**

This step describes the optimized protocol for the glutamate receptors staining and imaging by 3D-dSTORM. It may be applied to the staining of other surface membranes (Figure 1A).

***Note:*** Immunostaining was performed in free-floating 120 μm acute slices to increase antibody penetration.

19. Permeabilization.
  a. Incubate the slice with PBS – Triton (500 μL per slice) for 1 h at RT.
20. Block non-specific binding.
  a. Incubate the slice with PBS – Triton – 5% BSA for 1 h at RT. Troubleshooting 2.
21. Primary antibody incubation.
  a. Incubate the slice with PBS – Triton – 1% BSA containing the primary antibodies (200–500 μL per slice) with the appropriate dilutions (Table 1), ON at 4°C. Troubleshooting 1.
22. Wash unbound antibodies.
  a. Wash the slice three times with PBS – Triton – 5% BSA (500 μL per slice) for 15 min each at RT. Troubleshooting 2.
23. Secondary antibody incubation.
  a. Incubate the slice with PBS – Triton – 1% BSA containing the secondary antibodies (200–500 μL per slice) with the appropriate dilutions (Table 1), for 1–2 h at RT. Troubleshooting 1.
24. Wash unbound antibodies.
  a. Wash the slice three times with PBS – Triton or PBS (500 μL per slice) for 15 min each at RT. Troubleshooting 2.
25. Store in PBS at 4°C.

**CRITICAL:** Image in the following 2–3 days.

**CRITICAL:** Contrary to protocols for immunostaining for dSTORM of primary neuronal cultures do not perform a second fixation step, it may cause imaging artifacts.

### dSTORM acquisition

**Timing: 30 min per acquisition**

Sample mounting and dSTORM acquisition protocol for NMDARs (Figure 1B).

dSTORM relies on high stochastic fluorophore switching between different states, which requires proper sealing of the imaging chamber to maintain an oxygen-free environment. The pyranose switching buffer provides the reducing environment that allows the fluorophore to move from an “ON” bright state to an “OFF” dark state, and the enzymatic reaction acts as a scavenging oxygen system.

***Note:*** Imaging buffer composition may be adjusted to achieve a robust switching rate of the fluorophore.^14^

The fluorescence light is collected using a silicone immersion objective having an objective collar that was adjusted to minimize the optical aberration (spherical aberration) at the targeted imaging depth. The light is then detected by a SELFI camera in widefield to acquire images.

***Note:*** We predict the presented method can be used for the 3D-dSTORM imaging of other surface proteins, namely other neurotransmitter receptors, given that specific antibodies are available and that they are highly abundant in the tissue.

26. Organotypic brain slice mounting.
  a. Prepare the Ludin chamber with an 18 mm coverslip.
  b. Place the slice on top, with the slice facing the bottom.
  c. On top, place a piece of glass slice holder (cut with a diamond knife) to minimize the slice drift while keeping the light path transparent.
  d. Fill with switching buffer and close with a second coverslip to seal. Troubleshooting 3.

**CRITICAL:** Prepare the fresh switching buffer for every acquisition. Avoid air bubbles, any oxygen in the imaging chamber will compromise fluorophore switching and consequently the number of detections.

27. Acute brain slice mounting.
  a. Use a plain microscope slide with an imaging spacer of 120 μm.
  b. Add the slice in the middle of the imaging spacer.
  c. Fill with switching buffer.
  d. Seal with an 18 mm coverslip. Troubleshooting 3.

**CRITICAL:** Prepare the fresh switching buffer for every acquisition. Avoid air bubbles, any oxygen in the imaging chamber will compromise fluorophore switching and consequently the number of detections.

28. Acquisition of transmitted light to perform 3D autofocusing.
  a. Use transmitted light at 560 nm for 3D autofocus.
  b. Use a quantitative phase imaging module imaging the transmitted light to determine 3D drifts from image processing.
  c. Perform active axial stabilization by moving the microscope objective in real time to keep the sample in focus during acquisition.
  d. Use residual nanometric z-drifts and xy-drifts recordings and take them into account to determine the super-localization position of each molecule based on the initial image frame.

**CRITICAL:** Before proceeding to the dSTORM acquisition ensure the quality of the slices through visual inspection using transmitted light or phase imaging.

29. dSTORM acquisition.
  a. Use the 640 nm laser at 200 kW/cm^2^ to illuminate the sample.
  b. Record the fluorescence on a SELFI camera with 20 ms exposure time (typical fluorophore ON-time).
  c. Use a low amount of near UV light to control the blinking rate.

### Image reconstruction and analysis

**Timing: minimum 1 h, variable depending on the number of localizations per image and speed of processing computer**

Description of the image reconstruction and quantitative analysis of the number of nanodomains within NMDAR clusters.

Post-processing is subsequently performed to obtain single fluorescent emitter super-localization as demonstrated in^15^ and detailed below. For each frame, the background - calculated from a time-sliding average of images acquired before the frame in question is subtracted. Pre-localization of each single molecule in each background-free frame is performed using a kernel-based detection above a signal threshold. 3D super-localization is then calculated from sub-images cropped from the background-free SELFI image at each pre-localized position. For this purpose, the xy super-localization is obtained by conventional 2D Gaussian fitting after applying a low-pass filter to the sub-image to remove interference fringes and retain only the signal envelope, e.g., the conventional PSF response. In addition, the axial super-localization is obtained by comparing the interference image to a look-up table of a single fluorescent emitter response (100 nm nano-bead) as a function of defocus (acquired before the experiment). From all localization, detection filtering is performed to eliminate low signal levels and artifactual localization before reconstructing the super-localized images. For simplified visualization, z-color-coded 2D images were generated from each molecule localization (Figure 1C).

30. Single-molecule super-localization of each frame.
  a. Subtract the background.
  b. Perform the prelocalization of each detection.
  c. Retrieve the 3D super-localization of each prelocalized signal from the self-interference pattern.
31. Filter localizations to remove the poorly localized molecules (due to low signal level) and the localization artifacts (due to photon or instrumental noise). Troubleshooting 4.
32. NMDAR clusters segmentation.
  ***Optional:*** SR-Tesseler cluster identification is optional, other cluster segmentation software may be used. It is helpful for an unbiased definition of clusters, in highly dense SR images, as are hippocampal slices which express high amounts of NMDARs. Identification may be made by visual inspection of the reconstructed SR image only.
  a. Convert filtered localizations into the SR-Tesseler format.
  b. Export localizations into a .txt file.
  c. Import localization coordinates into SR-Tesseler.
  d. Create polygons on all detections using the Voronoi construction tab.
  e. Move to the object tab to segment the NMDAR clusters, by defining a density factor of 2, a minimum area of 2 × 104 nm^2^, corresponding to approximately 140 nm diameter clusters, and a minimum number of localizations of 5.
  f. Click “set density factor” followed by “Create objects”.
33. 3D-clusterization.
  a. Define the NMDAR nanodomains with the following criteria that are based on the dimensions of NMDAR (12 nm extracellular extension from the membrane and 14 nm diameter from an extracellular top view) and the previously described nanoscale organization of synaptic NMDAR in cultured neurons (2-5 nanodomains per synapse, ∼60 nm diameter nanodomain) :
    i. Minimal distance of 14 nm.
    ii. Maximal distance depending on the localization precision (typically 10 nm in xy and 20 nm in z) determined from the signal-to-background ratio, and the primary/secondary antibody size (5 nm).
      ***Note:*** A measurement via 3D Fourier ring correlation can be performed to actually determine the expected resolution and thus automatically set the maximal distance.
    iii. Minimal number of neighbors 3.
    iv. Minimum points 5.
      ***Note****:* Most of the parameters are automatically determined from physical measurements (number of photons, primary/secondary antibody size, actual resolution) and the minimal n# of points and neighbors is fixed either from biological knowledge or on varying them and monitoring the number of clusters to see a plateau in the value.

## Expected outcomes

To achieve the 3D position of the emitter, we combined conventional dSTORM microscopy with fluorescent self-interference (SELFI).^2^ SELFI relies on quantitative phase and intensity measurements and can be seen as the transposition to fluorescence microscopy of lateral shear interferometry, a well-established technique for quantitative phase microscopy. It has the advantage of introducing a negligible broadening of the point spread function (PSF) compared with other 3D localization methods, and of being only slightly sensitive to optical aberrations, including those introduced by the sample itself. 3D-dSTORM was first demonstrated in plated cells and 150 μm-thick tissue spheroids derived from human induced pluripotent stem cells (hIPSC).^2^ Previously, we had shown using cultured neurons that the nanoscale organization of NMDAR is dependent on the receptor’s subunit composition.^1^ We compared the 3D clustering with the corresponding 2D xy (in plane) projection and demonstrated that 3D-dSTORM captures on average 1 nanodomain more than the 2D projection.^1^ The number of nanodomains per cluster identified with 3D-dSTORM was comparable to the number of nanodomains captured by conventional dSTORM, and representative of the diversity of nanoscale organizations.^1^^,^^3^^,^^4^^,^^5^

Given the initial successful implementation of 3D-dSTORM by SELFI in tissue spheroids, we decided to extend its application to the use of native more complex cellular models like organotypic hippocampal slices and acute brain slices (Figure 1). The imaging procedure was performed as previously described.^2^ First, transmission light is used to identify by eyes the imaging region including depth within the sample. The collar of the imaging objective is set for the chosen depth to minimize spherical aberration due to sample thickness. Then an active nano-autofocusing process is initiated using the fixed sample itself as a reference.^15^ With this stabilization activated, dSTORM acquisition is performed in widefield for a few tens of minutes and imaged on a SELFI camera.

**Figure 2:**
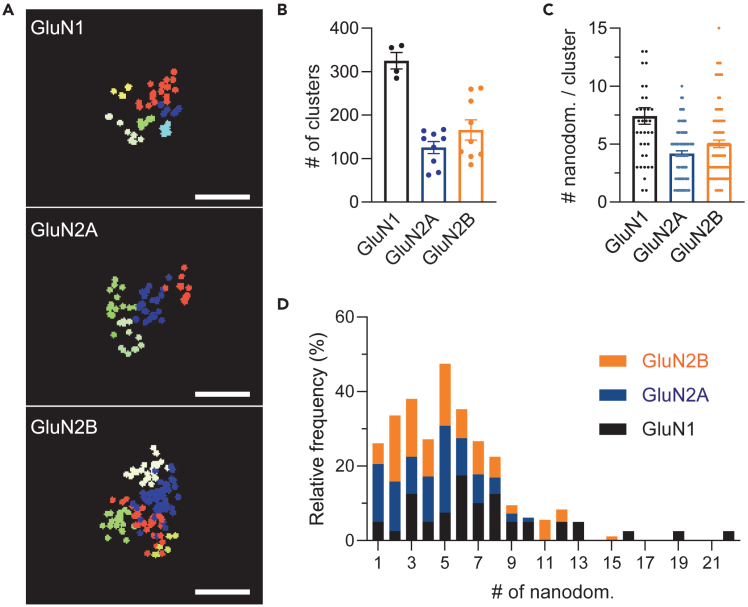
Nanoscale organization of NMDAR in organotypic hippocampal slices (A) Organotypic slices 10–12 div labeled with Anti-GluN1, GluN2A or GluN2B antibodies. Images prepared with LabVIEW, xz projections. Each segmented nanodomain within the selected cluster is represented by a different color, each point represents a detection. Scale bar: 200 nm (B) Number of clusters identified per imaging region (30 × 30 μm) from two to three independent experiments, one to three slices per experiment. Data presented as mean ± sem. GluN1 325 ± 19, GluN2A 125 ± 14 GluN2B 166 ± 23 clusters. (C) Number of nanodomains per cluster. Analysis of 10 clusters per slice. *N* = 40 GluN1, *N* = 90 GluN2A, *N* = 90 GluN2B. Mean ± sem: GluN1 7.4 ± 0.7, GluN2A 4.2 ± 0.2, GluN2B 5 ± 0.3 nanodomains per cluster. (D) Frequency distribution (percentage) of the number of nanodomains per cluster (bin center = 1).

**Figure 3:**
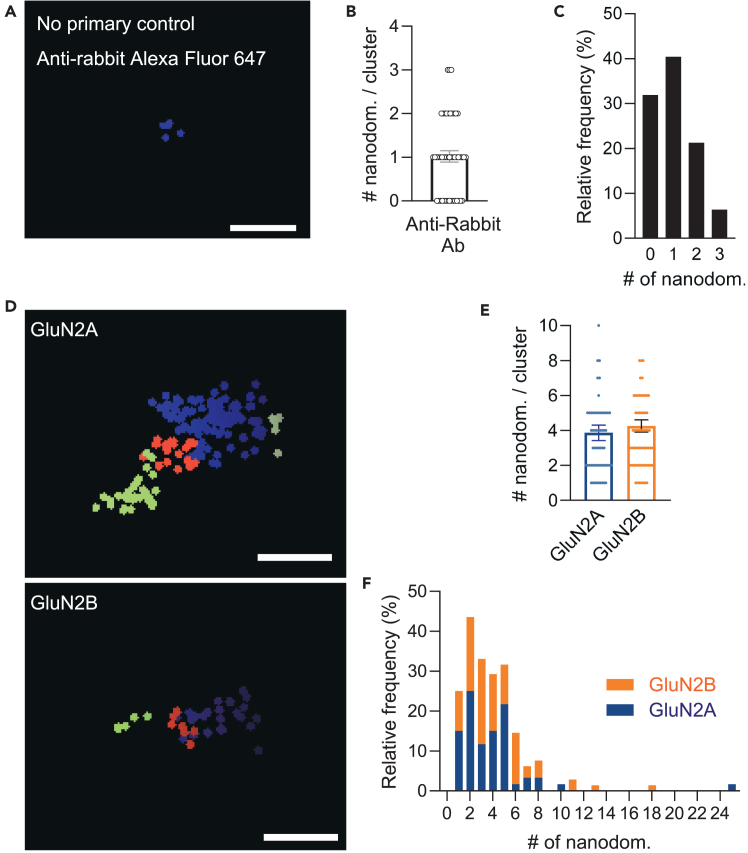
Nanoscale organization of NMDAR in acute brain slices (A) Acute brain slices from post-natal day (P)37 Wistar rats (*Rattus norvegicus*) labeled with anti-rabbit A647 (as control). (B) Number of nanodomains per cluster. Analysis of 47 clusters, from two brain slices. Data presented as mean ± sem.: 1 ± 0.1 nanodomains. (C) Frequency distribution (percentage) of the number of nanodomains per cluster (bin center = 1). (D) Acute brain slices from post-natal day (P)37 Wistar rats (*Rattus norvegicus*) labeled with Anti- GluN2A or GluN2B antibodies. Images prepared with LabVIEW, yz projections. Each segmented nanodomain within the selected cluster is represented by a different color, each point represents a detection. Scale bar: 200 nm (E) Number of nanodomains per cluster. Analysis of 10 clusters per slice. *N* = 60 GluN2A, *N* = 70 GluN2B, from six to seven slices. Data presented as mean ± sem. GluN2A 3.9 ± 0.4, GluN2B 4.3 ± 0.4 nanodomains per cluster. (F) Frequency distribution (percentage) of the number of nanodomains per cluster (bin center = 1).

Using this modified protocol for the labeling of NMDAR in brain slices, based on our previous experience using neuronal primary cultures^1^^,^^3^ and published protocols performing dSTORM with brain tissue,^11^^,^^16^ it is possible to successfully perform 3D-dSTORM in organotypic hippocampal slices (Figure 2) and acute brain slices (Figure 3). We started by validating the imaging in depth using 10–12 days *in vitro* (div) hippocampal organotypic slices, which are known to flatten over time (starting from 350 μm thickness when sliced to ∼200 μM after 5–10 div).^17^ On average, 27 nm xy- and 58 nm z-resolutions were thus obtained (mean ± sem: GluN1 xy 25 ± 1 nm, z 51 ± 4 nm; GluN2A xy 26 ± 1 nm, z 56 ± 4 nm; GluN2B xy 29 ± 3 nm, z 66 ± 9 nm). Using the SR-Tesseler segmentation approach,^7^ we were able to identify on average (mean ± sem) 325 ± 19 GluN1 clusters, 1254 ± 13 GluN2A-NMDAR clusters, and 166 ± 23 GluN2B-NMDAR clusters (Figure 2B). The higher percentage of GluN1 clusters is consistent with the fact that GluN1 is the obligatory subunit of NMDAR. The marginally bigger number of GluN2B-containing NMDAR points towards an immaturity of organotypic slices. We selected 10 clusters per slice and analyzed the 3D-nanoclusterization using homemade routines developed in LabVIEW (National Instruments). The algorithm associates super-localized detections if their inter-distances are close enough. Given the precision of 3D localization and the dimensions of the primary/secondary antibody complex, we also distinguished clusters from multiple detections of the same receptor when detections were too close together.

As a control, we imaged five slices incubated with secondary antibodies only (one incubated with the anti-mouse A647, and four with anti-rabbit A647). As expected the washes were not fully efficient. For anti-mouse A647 we could identify 77 clusters and for anti-mouse A647 we identified 50 clusters, which is well under the number of clusters identified in the specific labeling. Moreover, when we analyzed their nanodomain composition we found a very reduced number of nanodomains (Figure S1). Additionally, the resolution of these images, both in xy and z, was substantially poorer than resolutions obtained in the experimental conditions (mean ± sem: 42 ± 10 nm in xy, 98 ± 22 nm in z). We found NMDAR clusters in organotypic slices are organized into a wide range of nanodomains numbers (Figure 2C), from only one nanodomain (less than 5%) to sporadically 22 nanodomains (Figure 2D). The majority of NMDAR (65%) has between three to eight nanodomains. As for primary neuronal cultures,^1^ different nanodomains composition can be identified depending on the subunit composition (Figure 2C). While GluN2A-containing NMDAR (GluN2A-NMDAR) major population is composed of five nanodomains (23%), GluN2B-containing NMDAR (GluN2B-NMDAR) most abundant population is composed of two nanodomains (18%). As previously suggested, GluN2B-NMDAR composition seems more plastic and dynamic,^1^^,^^3^ we find a wider range of nanodomain numbers in GluN2B-containing clusters (1 to 15 nanodomains), the majority ranging from two to five nanodomains, compared with GluN2A-containing (1 to 10 nanodomains).

Similarly, we found NMDAR organized into multiple nanodomains in acute brain slices (Figure 3). Resolution of obtained images was on average 21 nm in xy and 43 nm in z (mean ± sem: GluN2A 21 ± 1 nm in xy, 45 ± 1 nm in z; GluN2B 20 ± 1 nm in xy, 41 ± 4 nm in z). Similarly to organotypic brain slices, the resolution of the negative control images was poorer than the experimental conditions (xy 51 ± 4 nm, z 113 ± 6 nm). Using acute brain slices from postnatal day pups 37 (P37), we found on average four nanodomains per cluster (mean ± sem: 3.7 ± 0.4 GluN2A-NMDAR, 4.3 ± 0.4 GluN2B-NMDAR). As in organotypic brain slices, NMDAR in acute brain slices presents a heterogeneous composition (Figures 3D–3F), from one to 25 nanodomains for GluN2A-NMDAR and 1 to 18 nanodomains for GluN2B-NMDAR. In the negative control, using only the secondary antibody, within the 47 clusters analyzed from six slices, the majority (72%) either presented no nanodomains or only one (Figures 3A–3C).

**Figure 4:**
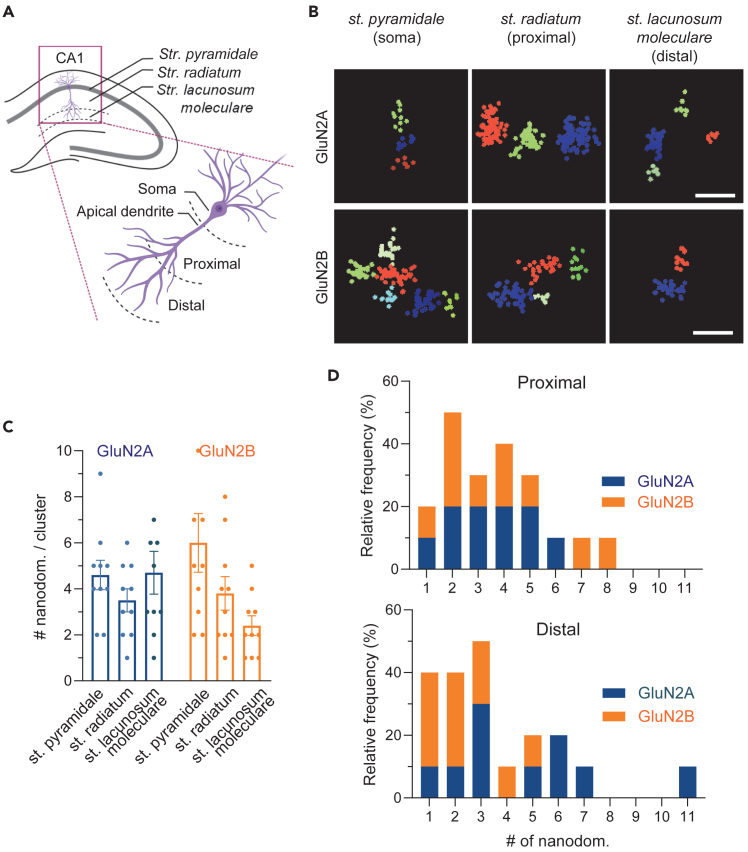
Nanoscale organization of NMDAR along the apical dendrite (A) Imaging of CA1 region, at three different locations: *Stratum (St.) pyramidale, St. radiatum, St. lacunosum moleculare*, corresponding to soma layer, apical proximal dendrites and apical distal dendrites, respectively. (B) Acute brain slices from P17 Wistar rats (*Rattus norvegicus*) labeled with Anti-GluN2A or GluN2B antibodies. Images prepared with LabVIEW, xz (GluN2A) and yz (GluN2B) projections. Each segmented nanodomain within the selected cluster is represented by a different color, each point represents a detection. Scale bar: 200 nm (C) Number of nanodomains per cluster. Analysis of *N* = 10 clusters per layer from one slice. Data presented as mean ± sem. GluN2A 4.6 ± 0.6 *St. pyramidale*, 3.5 ± 0.5 *St. radiatum*, 4.7 ± 0.9 *St. lacunosum moleculare*, GluN2B 6 ± 1.3 *St. pyramidale*, 3.8 ± 0.7 *St. radiatum*, 2.4 ± 0.4 *St. lacunosum moleculare*. (D) Frequency distribution (percentage) of the number of nanodomains per cluster (bin center = 1).

Recently, our group showed, using primary hippocampal cultures, that GluN2B-NMDAR nanoscale organization highly changed according to the distance to the cell body, while GluN2A-NMDAR were relatively stable.^3^ Using 3D-dSTORM we analyzed the composition of NMDAR clusters along the apical dendrites of pyramidal cells on the CA1 region of the hippocampus in P17 acute brain slices (Figures 4A and 4B, mean ± sem xy and z resolution: GluN2A in xy, 23 ± 0.4 nm and 45 ± 1 nm in z; GluN2B in xy, 28 ± 1 nm and 50 ± 3 nm in z). The hippocampus is organized by well-defined regions and layers.^18^ We focused on the CA1 area and imaged sequentially in the *stratum (st.) pyramidale*, where pyramidal neurons soma are concentrated, the *st. radiatum* where we find apical dendrites proximal to the soma (between 30 to 120 μm) and finally the *st. lacunosum moleculare* populated by the apical dendrites distal to the soma (>120 μm) (Figure 4A). Interestingly, as in primary cultures, we observed that GluN2A-NMDAR was more homogenously distributed along the apical dendrite: *st. pyramidale* (soma) 4.6 ± 0.6, *st. radiatum* (proximal apical dendrites) 3.5 ± 0.5, *st. lacunosum moleculare* (distal apical dendrites) 4.7 ± 0.9 nanodomains per cluster (Figures 3B and 3C). On the contrary, GluN2B-NMDAR nanodomain composition highly changed with the distance to the neuron soma [mean ± sem: *st. pyramidale* (soma) 6.0 ± 1.3, *st. radiatum* (proximal apical dendrites) 3.8 ± 0.7, *st. lacunosum moleculare* (distal apical dendrites) 2.4 ± 0.4 nanodomains per cluster]. We observed the number of GluN2B-containing nanodomains highly decreased with the distance to the cell soma (Figures 4B and 4C). Note that 60% of GluN2B-NMDAR clusters in distal apical dendrites are composed of only one or two nanodomains, while the composition of the clusters in proximal apical dendrites was more heterogenous (Figure 4D). This data confirms that the nanoscale organization of NMDAR highly contributes to the synapse function diversity along the hippocampal neuron dendrites.^3^

In summary, our protocol establishes that NMDARs, composed of either the GluN2A or the GluN2B subunits, are in the vast majority composed of more than two nanodomains. Our observations show that the nanoscale organization of NMDAR in brain slices is as diverse and regulated as we had previously observed in neuronal cultures,^1^^,^^3^ which is probably true for other synaptic proteins and glutamate receptors. The implementation of this protocol will pave the way for the understanding of the receptor’s nanoscale organization function for synapse physiology, by allowing for example the visualization of nanodomains after neuronal activity measurements and/or manipulation. Moreover, it is now possible to understand if synaptic nanodomains may differ depending on the brain regions or the types of synaptic contacts.

## Quantification and statistical analysis

For comparison of the nanoscale composition of NMDAR clusters in brain slices, super-resolved reconstructed images were segmented with SR-Tesseler,^7^ for unbiased segmentation of NMDAR clusters. Alternative segmentation software is available,^19^ e.g., DBSCAN. Segmentation parameters were based on our previous experience with neuronal cultures.^1^^,^^3^ NMDAR clusters were selected based on the high density of localizations, with a minimum size of approximately 140 nm and with at least five localizations, the minimum number to draw a polygon. Analysis parameters may be adjusted for the analysis of other surface proteins, according to their size and/or previously described distribution. Ten clusters were randomly selected to be further segmented into nanodomains. The clustering algorithm considered the 3D localization precision of each single molecule (on average 10 nm in xy for 20 nm in z) and the primary/secondary antibody bulkiness (∼5 nm) to define an influence zone around each localization. If two influence spheres shared a common volume, the molecules were considered in the same cluster. After considering all possible associations, each generated cluster was fitted with a 3D ellipsoid to determine its characteristics (central position, 3D orientation, and lengths). Points closer than the apparent dimension of one receptor (∼14 nm, i.e., its influence zone) were merged and considered as a double detection of the same receptor.

NMDAR nanodomains were defined as high-density areas, within the selected clusters, composed of at least three neighboring localizations that were less than 14 nm distance, corresponding to approximately the size of one tetramer, and composed of at least five localizations. Analysis parameters may be adjusted according to the size of the protein being studied.

## Limitations

The present protocol provides the protocol for labeling NMDARs on brain slice models (organotypic and acute) and performing dSTORM to characterize the nanoscale organization of these synaptic receptors. Given the wide expression of NMDARs in hippocampal brain slices, at least 100 NMDAR clusters should be identifiable in a super-resolved image of 30 × 30 μm. For a good segmentation of the NMDAR cluster, a quasi-isotropic resolution (i.e., xy of 25 nm and a z resolution of 50 nm) should be achieved, to account for different synaptic orientations.

NMDARs have a well-known role in synapse establishment,^20^^,^^21^ being recruited to synaptic localizations early in development. In young and adult brain slices, NMDAR are expected to be mainly synaptic.^22^ Therefore, a significant portion of the clusters analyzed using this protocol are expected to be synaptic. To specifically study synaptic NMDARs, a synaptic marker like PSD95 can be used.^3^

dSTORM cannot predict the absolute number of receptor complexes within the nanodomains. Because NMDARs may be composed of one or two GluN2A or GluN2B subunits,^23^ the labeling with the primary antibody followed by the secondary antibody may not follow a 1:1 ratio. Additionally, due to the stochastic nature of the fluorophores, there is the possibility that some receptor clusters are not detected, or the detection is excluded due to poor photon count. If labeling and imaging protocols are consistently maintained across experimental conditions, the detection density can be quantitatively compared and utilized as an indicator of receptor complex abundance.

As for *in vitro* cultures, the major limitation of this protocol is the availability of primary antibodies that specifically recognize the protein of interest and the abundance of the protein, which will limit the density of localizations and consequently the resolution that can be achieved.^24^ We strongly advise when setting up this protocol performing control experiments to evaluate antibody specificity e.g., probing the antibody using a knock-out model. Due to the reconstructive nature of the imaging method, it is prone to artifacts.^25^ For this reason, extra attention should be given to the sample preparation, therefore experiments without primary antibodies should be run in parallel, as in Figures S1 and 4A–4C to probe for imaging artifacts arriving from non-specific labeling of secondary antibodies. The amount of data generated, especially in 3D, renders the analysis quite time-consuming and currently very user-dependent. In the future automation of the analysis will be crucial to fully process all the acquired data.

Brain preparation requires users to have specific formations on animal experimentation and to follow specific regulations in place for animal use. Researchers with previous experience in brain slice preparation and light fluorescent microscopy are recommended. Experience using LabVIEW software (or equivalent software) will be essential for customizing imaging acquisition and analysis.

## Troubleshooting

### Problem 1

Poor staining may be due to poor penetration or insufficient number of antibodies (related to steps 6, 11, 21, and 23).

### Potential solution

One should first increase incubation times or antibody concentrations that are used to stain the target, i.e., the NMDAR. If the problem remains, probe the cell viability of your sample and/or use another antibody that has been previously validated.

### Problem 2

High non-specific signals may be due to the non-specific binding of primary or secondary antibodies (related to steps 7, 9, 10, 12, 20, and 24).

### Potential solution

• Increase BSA or gelatin percentages on the blocking step or increase the washing step duration to remove all unbound particles.
• A negative control without the primary antibody should be performed for each secondary antibody subtype (Figure S1) for specificity control.
• Use pre-coupled primary-secondary antibody complexes to reduce the putative noise induced by each of the antibody alone.

### Problem 3

The low fluorophore switching rate may be due to oxygen in the imaging chamber from prolonged imaging sessions or the entry of air bubbles (related to steps 26 and 27).

### Potential solution

Prepare fresh pyranose switching buffer and/or reseal imaging chamber.

### Problem 4

Poor resolution of the reconstructed image may be due to a low number of detected localizations due to poor sample preparation or an insufficient number of acquisitions (related to step 31). Thanks to the resolution measurement within the software (using 3D ring correlation), an easy go/no-go analysis as well as performing an auto-tuning of the clustering distance in order to avoid over or under clustering. When the evaluated resolution is much degraded (>50 nm in lateral), the acquisition is considered as bad.

### Potential solution

Review sample preparation protocol or increase acquisition time, respectively.

## Resource availability

### Lead contact

Further information and requests for resources and reagents should be directed to and will be fulfilled by the lead contact, Laurent Groc (laurent.groc@u-bordeaux.fr).

### Technical contact

Technical questions on executing this protocol should be directed to and will be answered by the technical contact, Joana S. Ferreira (joana.s.ferreira@uc.pt).

### Materials availability

This study did not generate new unique reagents.

### Data and code availability

The datasets/code generated during this study is available upon request to the lead contacts.

## Acknowledgments

This work was performed with financial support from the European Research Council Synergy grant (951294), 10.13039/501100001665Agence Nationale de la Recherche (ANR-15-CE16-0004-03), and the France-BioImaging National Infrastructure (ANR-10-INBS-04-01). We would like to thank the Cell Biology Facility, especially Delphine Bouchet and Emeline Verdier, for molecular and cellular tool productions and the IINS *in vivo* facility for animal housing. Animal experiments were performed at the Animal Facilities of the University of Bordeaux. J.S.F. acknowledges funding from the Portuguese Foundation for Science (FCT), project reference 2020.01761.CEECIND/CP1609/CT0003.

## Author contributions

J.S.F., J.L.-L., and P.B. planned and carried out the experiments; P.B. designed the homemade analysis software; J.S.F. and J.L.-L. analyzed the data; J.S.F., P.B., L.C., and L.G. designed the study; L.C. and L.G. secured the funding; and J.S.F., P.B., L.C., and L.G. wrote the manuscript. All authors discussed the data and commented on the manuscript.

## Declaration of interests

The authors declare no competing interests.
