# Supplementary material for: Protocol for performing 3D-STORM-based nanoscale organization of NMDA receptors in hippocampal brain tissue
Source: STAR Protoc. 2025 Feb 24;6(1):103639. doi: 10.1016/j.xpro.2025.103639 (PMC11903812; doi:10.1016/j.xpro.2025.103639)
Supplement: Document S1. Figures S1 [file mmc1.pdf]

Figure S1

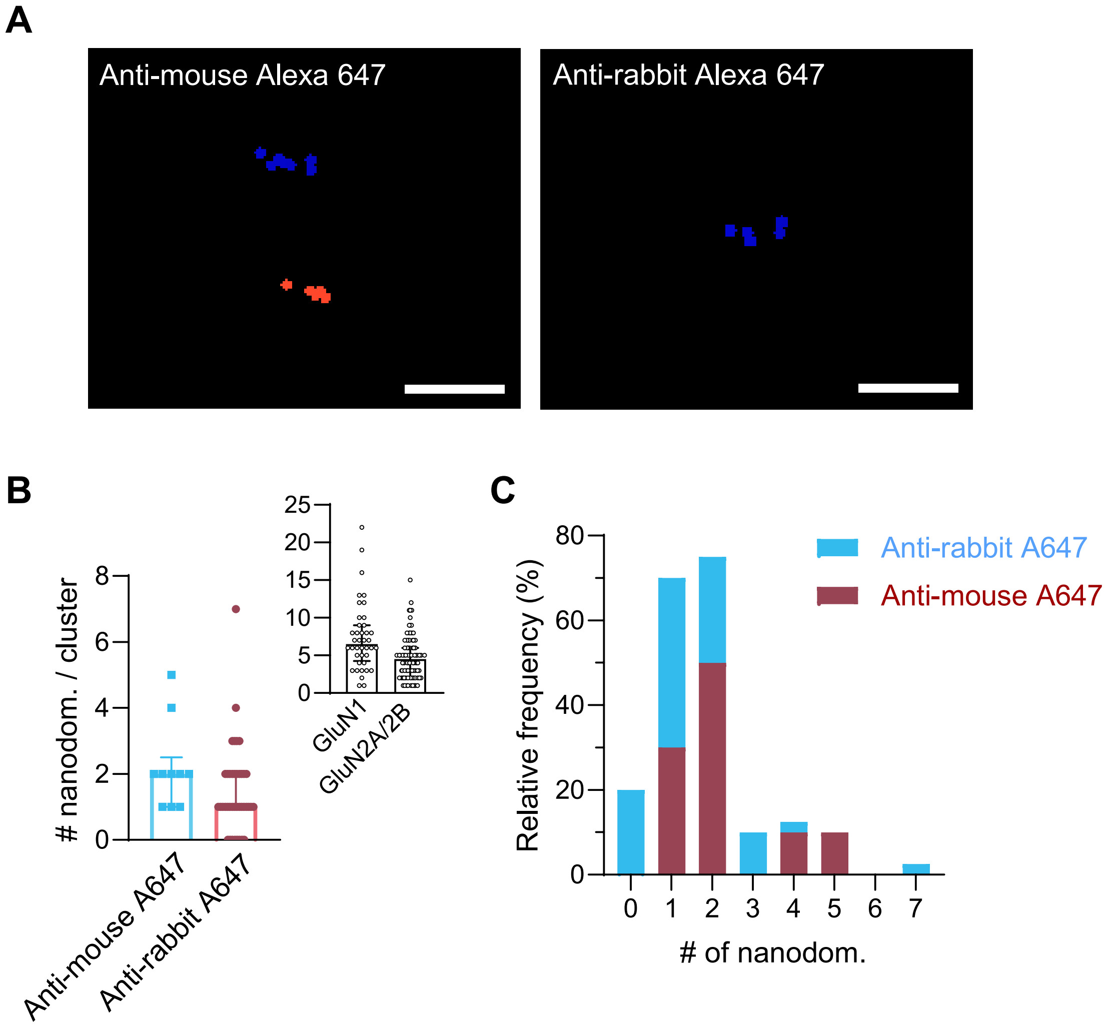

Figure S1 (related to Figure 2): **Control staining to be performed in organotypic slices.** (A) Organotypic slices 10-12 div labeled with Anti-mouse A647 and Anti-rabbit A647 secondary antibodies only. Images prepared with LabVIEW, yz projections. Each segmented nanodomain within the selected cluster is represented by a different color, each point represents a detection. Scale bar: 200 nm. (B) Number of nanodomains per cluster. Analysis of 10 clusters per slice. *N*=10 Anti-Mouse A647, *N*=40 Anti-Rabbit A647 nanodomains per cluster. Data presented as mean ± sem. Anti-Mouse A647 2.2 ± 0.4, Anti-Rabbit A647 1.5 ± 0.2 nanodomains/cluster. **i)** Number of nanodomains per cluster presented in Fig. 2C, GluN2A and GluN2B nanodomains pulled into one column. Data presented as mean ± sem. GluN1 7.4 ± 0.7, GluN2A/2B 4.6 ± 0.2 nanodomains/cluster. (C) Frequency distribution (percentage) of the number of nanodomains per cluster (bin center = 1).
